# Crystal Structure of Hcp from *Acinetobacter baumannii*: A Component of the Type VI Secretion System

**DOI:** 10.1371/journal.pone.0129691

**Published:** 2015-06-16

**Authors:** Federico M. Ruiz, Elena Santillana, Mercedes Spínola-Amilibia, Eva Torreira, Esther Culebras, Antonio Romero

**Affiliations:** 1 Department of Chemical and Physical Biology, Centro de Investigaciones Biológicas—CSIC, Madrid, Spain; 2 Department of Clinical Microbiology, Hospital Clínico San Carlos, Madrid, Spain; Centre National de la Recherche Scientifique, Aix-Marseille Université, FRANCE

## Abstract

The type VI secretion system (T6SS) is a bacterial macromolecular machine widely distributed in Gram-negative bacteria, which transports effector proteins into eukaryotic host cells or other bacteria. Membrane complexes and a central tubular structure, which resembles the tail of contractile bacteriophages, compose the T6SS. One of the proteins forming this tube is the hemolysin co-regulated protein (Hcp), which acts as virulence factor, as transporter of effectors and as a chaperone. In this study, we present the structure of Hcp from *Acinetobacter baumannii*, together with functional and oligomerization studies. The structure of this protein exhibits a tight β barrel formed by two β sheets and flanked at one side by a short α-helix. Six Hcp molecules associate to form a donut-shaped hexamer, as observed in both the crystal structure and solution. These results emphasize the importance of this oligomerization state in this family of proteins, despite the low similarity of sequence among them. The structure presented in this study is the first one for a protein forming part of a functional T6SS from *A*. *baumannii*. These results will help us to understand the mechanism and function of this secretion system in this opportunistic nosocomial pathogen.

## Introduction

Pathogenic bacteria use macromolecular protein complexes (1 to 20 molecules), known as secretion systems, in order to transport toxic proteins into the environment or directly into neighboring target cells. In the last decade, several types of secretion systems have been described in Gram-negative bacteria [1, 2]. The most recently discovered is the Type VI Secretion System (T6SS), initially described in *Vibrio cholerae* in 2006 [3]. This protein translocation pathway was originally shown to involve a cluster of around 15 genes. Subsequently, a T6SS was characterized in *Pseudomonas aeruginosa*, together with the first structure of a protein from this complex [4]. In *P*. *aeruginosa* the T6SS delivers toxins to other bacteria, showing that this secretion system is not only used to attack host cells but also, to compete with other bacteria in the same niche. Accordingly, it has been proposed that the T6SS may serve to control the composition of bacterial populations, an important issue in polymicrobial infections [5, 6].

T6SS gene clusters are normally found in pathogenicity islands and they were not acquired by lateral genes transfer [7]. Thirteen proteins form the core of a functional T6SS mimicking a bacteriophage spike anchored to to the bacterial membrane via a transmembrane complex [2, 6, 8]. According to the nomenclature adopted by Shalom *et al*. [9] the core components are called Tss (Type six secretion), while accessory subunits are called Tag. Vernacular nomenclature is kept for some subunits such as Hcp, VgrG, ClpV, VipA and VipB. Hcp (haemolysin conjugated protein) and VgrG (valine-glycine repeat protein G), products of the genes *tssD* and *tssI*, are the homologues of the bacteriophage tail gp19 and the tail spike complex (gp27)_3_-(gp5)_3_, respectively [1, 2]. Based in this analogy, these proteins are thought to be molecular markers of a functional T6SS and they may form the needle of this secretion system [8]. While the full-length structure of VgrG has not been solved yet, the crystal structure of five Hcp proteins have been reported: Hcp1 [4] and Hcp3 [10] from *P*. *aeruginosa*, EvpC from *Edwardsiella tarda* [11], an unpublished hypothetical Hcp protein from the T6SS of *Yersinia pestis* and, more recently, double mutant of Hcp1 from enteroaggregative *Escherichia coli* (EAEC) [12]. Significantly, all of them are composed by a β-barrel forming hexameric rings oligomers with a diameter that can accommodate small folded (< 20 kDa) or unfolded proteins [8]. Small effectors, such as EvpP in *E*. *tarda* [13] and Tse1-3 in *P*. *aeruginosa*, directly interact with Hcp proteins. In particular, these last three proteins bind to the inner surface of the Hcp hexamer, as shown by mutational studies and negative-staining electron microscopy. In addition, the increased stability of Tse1-3 effectors in presence of Hcp suggests a role of chaperone for this protein [14].

The Gram-negative bacillus *Acinetobacter baumannii* is an opportunistic pathogen that has raised the attention of authorities due to its ability to develop multi-drug resistance and to acquire genetic material from unrelated genera [15, 16]. In addition, *A*. *baumannii* can form biofilms and survive for weeks in dry environments, contributing to endemic infections in healthcare installations [17]. The number of infections caused by this coccobacillus has increased dramatically in recent years, mainly in inmunocompromised or ventilator-dependent patients who suffer prolonged hospitalization. Its most frequent clinical manifestations include soft tissue, urinary tract and blood stream infection, meningitis and pneumonia [18, 19]. Most scientific research about *A*. *baumannii* has focused on its epidemiology and on antibiotic resistance. As result, it is only recently that the mechanisms of virulence employed by this bacillus have begun to be identified. Among these, a conserved protein glycosylation system plays an important role in pathogenicity and film formation [20]. Moreover, the OmpA outer membrane protein plays a key role in the interaction with epithelial cells at the early stages of infection [21], as well as participating in biofilm formation. In addition the *A*. *baumannii* Omp38 protein has been localized within the host cell mitochondria, inducing apoptosis of epithelial cells [22].

As described in 2013, many species within the *Acinetobacter* genus (including non-pathogenic ones) have a conserved and functional T6SS that contains homologues of 12 core T6SS genes, including the hallmarks *tssD* (Hcp), *tssH* (ClpV) and *tssM*. By contrast, the putative VgrG proteins are encoded by a varying numbers of genes located outside the T6SS loci [23]. The activity and function of the T6SS in *A*. *baumannii* have been recently studied in diverse strains. The Hcp protein was found in the culture supernatant of wild type ATCC 19606, confirming the functionality of the T6SS [24].

The *A*. *baumannii* strain M2 produces a functional T6SS and it uses it to diminish *E*. *coli* colony formation in a cell-cell contact and *tssB*-*tssD*-dependent manners. This behavior is similar to that reported for *Serratia marcescens*, *V*. *Cholerae* and *P*. *aeruginosa*, confirming the key function of the *A*. *baumannii* T6SS when competing with other bacteria [25].

In contrast, the T6SS of *A*. *baumannii* ATCC 17978 does not seem to be implicated in interbacterial killing. Nevertheless, a mutant of this strain harboring an inactive T6SS retained its virulence and capacity to form biofilms similar to that of the wild-type bacteria. These results suggest that the T6SS could fulfill another unidentified role in this coccobacillus [23].

In this study we show that the T6SS is active in six pathogenic strains of *A*. *baumannii*. We also present the structure of Hcp from *A*. *baumannii* AB0057 at 1.55 Å resolution, characterizing its self-interactions and oligorimezation. This structure resembles those defined previously for other members of the Hcp family, oligomerizing in donut-shaped hexamers with an inner diameter of 40 Å. However, the crystal packing of Hcp from *A*. *baumannii* is unique among these, forming a tube but interacting in both head-to-head and head-to-tail geometry. The structural information obtained could help us to understand the mechanism and function of this secretion system in this opportunistic nosocomial pathogen.

## Materials and Methods

### Functional assay of the T6SS

Single bacterial colony was added to 2 ml Muller-Hinton broth (MHB) and the cultures have grown for 15–18 h in 37°C rocking water bath (160 rpm). A total 500 μl of the overnight culture was added to 5 ml of pre-warmed MHB and incubated for 2 h on a shaker bath until turbidity reached a 1 McFarland standard (approximately 2.5x10^8^ CFU ml^-1^). An aliquot of this logarithmic-phase growth culture was used to colony counts. Aliquots (0.1 ml) from each sample were removed before centrifugation, serially diluted, spread on MH plates and incubated at 37°C. Bacterial colonies were counted after 18–24 h. Inoculum was between 2–5 x10^8^ CFU ml^-1^ in all samples. Cells were harvested by centrifugation, from 10 ml bacteria culture, to isolate the extracellular proteins, and then resuspended in 200 μl of SDS protein sample buffer and heated. TCA (250 μl) was added to 750 μl of the supernatant and the proteins were precipitated for 10 min on ice, after which they were recovered by centrifugation at 14000 g for 10 min. The precipitated proteins were washed twice with cold acetone, and then resuspended in 20 μl SDS protein sample buffer and boiled. Alternatively, the cleared supernatants were concentrated up to 200 μl by filtration using Amicon Ultra 10,000 MCWO centrifugal filter units (Millipore), 5 μl from this concentrated sample were mixed with 15 μl SDS loading buffer. All the protein samples were loaded into 15% polyacrylamide gels and transferred to PVDF membranes. The membranes were probed with polyclonal rabbit anti-Hcp (diluted 1:20,000) and anti-RNAP antibodies (diluted 1:20,000, *E*. *coli* RNA polymerase). Membranes were then incubated with an anti-rabbit horseradish peroxidase-conjugated secondary antibody (diluted 1:20,000; Sigma). Membranes were developed using ECL reagent (GE Healthcare) and visualized using a Fuji LAS 3000 imager.

DNA was isolated from all the samples in order to characterize their Hcp proteins by sequencing.

### Cloning, expression and purification

Genomic DNA isolated from the *A*. *baumannii* strain AB0057 was used to clone the full-length Hcp. Cloning was directed by the sense primer 5'-ACA**GCTAGC**ATGAAAGATATATACGTTGAG containing an internal NheI restriction site (bold) and the anti-sense primers 5'-ACA**CTCGAG**TTACGCTGCGTAAGAAGCTGT or 5'-ACA**CTCGAG**CGCTGCGTAAGAAGCTGTATT including an internal XhoI restriction site (bold), with or without the stop codon (underlined). The amplified PCR product was digested with NheI and XhoI (NEB), and ligated into the linearized pET21-a plasmid (Novagen) or a modified pET28-a (Novagen) plasmid that includes a human rhinovirus 3C protease cleavage site (3C-pET28). The recombinant plasmids were transformed into competent *E*. *coli* DH5α cells (Novagen) for DNA production and purification, and the integrity of both constructs was verified by sequencing. Finally, they were transformed into Rosetta pLys-S cells (Novagen) to express the protein. After induction with 1 mM IPTG, 1 l culture was grown at 30°C for 4 h. The bacterial cells recovered by centrifugation were disrupted by sonication and ultracentrifuged. The soluble fraction was loaded into a His-trap column (GE Healthcare) equilibrated with 350 mM NaCl, 20 mM Tris-HCl [pH 7.5], 1 mM βME and 20 mM Imidazol. The protein was subsequently eluted from the column with buffer containing Imidazol at a concentration of 250 mM. In the case of the Hcp-3C-pET28 construct, the eluted protein was incubated with 3C protease overnight at 4°C and loaded onto a His-trap column in order to remove the protease, the His-tag and the uncut sample. For both constructs the Hcp solution was finally concentrated and injected onto a Sephacryl S200 16/60 gel filtration column (GE Healthcare) equilibrated with a buffer containing 200 mM NaCl, 20 mM Tris-HCl [pH 7.5], 2 mM DTT and 5% w/v Glycerol. Both Hcp protein-constructs eluted in a single peak and the pooled peak fractions were concentrated to 20 mg ml^-1^ using Amicon Ultra 10,000 MCWO centrifugal filter units (Millipore) for crystallization trials. The purified product was analyzed by SDS-PAGE.

### Crystallization, data collection and processing

The first crystallization trials were set up at 4 and 22°C in 96-well sitting-drop plates (Swissci MRC) using a Cartesian Honeybee System (Genomic Solutions) and the JBS 1 to 8 commercial kits (Jena Bioscience). The nanodrops were 0.4 μl in size, containing 0.2 μl of the protein solution at 20 mg ml^-1^ and 0.2 μl of precipitant. They were equilibrated against 50 μl of the reservoir solution. Initial hit conditions were further optimized in sitting drops prepared by mixing 1 μl of protein solution and 1 μl of reservoir solution. Large crystals for both constructs were obtained in 2.0 M Ammonium Sulfate and 0.1 M Hepes [pH 7.5] at 4°C. These hexagonal prism crystals were soaked for 5 min in the reservoir solution supplemented with 1.0 M Na Malonate as cryoprotectant. For data collection, the crystals were flash-cooled by immersion in liquid Nitrogen. Diffraction data were collected at 100 K at beamlines PROXIMA-1 of the SOLEIL Synchrotron (France) and BL13-XALOC of the ALBA Synchrotron (Spain). The crystallographic data were processed using XDS [26] and Aimless [27]. A summary of the data collection and processing statistics is given in Table 1.

**Table 1:** Data collection and refinement statistics.

Wavelength (Å)	0.980110
Oscillation range (°)	0.2
No. of images	1000
Space group	P 6
Unit cell (Å)	a = b = 87.00; c = 128.81
Resolution range (Å)	48.96–1.55 (1.60–1.55)
Total No. of reflections	867266 (32649)
Unique reflections	79939 (7835)
Multiplicity	10.8 (8.3)
Completeness (%)	99.80 (98.06)
Rpim	0.026 (0.171)
Mean I/sigma(I)	20.8 (1.8)
Mn(I) half-set correlation CC(1/2)	1.00 (0.70)
Wilson B-factor	20.62
Molecules per asymmetric unit	3
Solvent content (%)	52
R-factor	0.1681 (0.2520)
R-free	0.1977 (0.2744)
Number of atoms	4522
macromolecules	3989
ligands	20
water	513
Protein residues	497
R.m.s.d. (bonds)	0.006
R.m.s.d. (angles)	1.09
Ramachandran favored (%)	97
Ramachandran outliers (%)	0
Clashscore	5.21
Average B-factor	25.30
Macromolecules	23.70
Solvent	36.50
PDB code	4W64

Data collection and refinement statistics for the Hcp structure. Statistics for the highest-resolution shell are shown in parentheses

### Structure determination and refinement

The structure of Hcp was solved through Molecular Replacement using a poly-Ala model of Hcp1 (PDB entry 1Y12 [4]) as the search probe. Translation-Libration-Screw (TLS) refinement was performed with the Phenix software suite [28] and TLS groups were defined using the TLMSD web server [29]. Manual building and water molecules placement was carried out with Coot [30]. Details of the model refinement are given in Table 1. The stereochemical validation of the final model and the analysis of protein-protein interactions were performed using the Molprobity [31] and PISA [32] web servers, respectively. Amino acid conservation was analyzed using the ConSurf server (http://consurf.tau.ac.il) and the electrostatic properties were studied using the APBS package [33]. Structural figures were drawn using Pymol [34].

### Analytical Ultracentrifugation

In order to avoid undesirable effects of the His-tag in the oligomerization studies, we have used the tag-free Hcp sample. The protein solutions at 0.016, 0.2, 1, 2.5, 8 and 20 mg ml^-1^ were prepared in a buffer containing 20 mM Tris–HCl, [pH 7.5], 200 mM NaCl, 0.2 mM DTT and 2% glycerol. Sedimentation velocity profiles were measured at 20°C on an Optima KL-I (Beckman) ultracentrifuge, with a Beckman An-50 Ti rotor and standard double-sector Epon-charcoal center pieces (1.2 cm optical path length). Sample and reference solutions were loaded and sedimented at 185,460 x g, registering successive entries every minute. Rayleigh interferometric detection was used to monitor the evolution of the concentration gradient in function of time and radial position, and the data were analyzed using the SedFit software (Version 12.52)[35]. Sedimentation equilibrium experiments were performed at 20°C on an Optima XL-A (Beckman) ultracentrifuge equipped with UV-visible absorbance optics using an An-50 Ti rotor. Short-column (85 μl) sedimentation equilibrium runs were carried out at 5,150 and 8050 x g using absorbance scans at 250 and 290 nm. The weight-average buoyant molecular mass was calculated using the Hetero-Analysis program (Version 1.1.44)[36], and the molecular mass was determined using a value of 0.7300 cm^3^ g^-1^ for the partial specific volume and 1.01150 g cm^-3^ for the density.

### Electron microscopy

The tag-free Hcp sample was diluted to 0.02 mg ml^-1^ in a solution of 20 mM Tris–HCl [pH 7.5], 200 mM NaCl and 2 mM DTT, and stained with 1% uranyl formate. Electron micrographs were recorded on a JEOL 1230 electron microscope operated at 100 kV. Images were collected using a 4k x 4k CMOS detector under the control of the EM-Tools software (TVIPs). The final magnification of each micrograph was 68222.5x. 4800 Individual particles were manually selected using the package Boxer implemented in EMAN 1.9. 2D reference-free classification was achieved using the EMAN 1.9 and Xmipp [37, 38].

## Results

### *Acinetobacter baumannii* strain AB0057 has a functional T6SS

Analysis of the genome of the *A*. *baumannii* strain AB0057, isolated from a bloodstream infection, allows identifying the presence of genes encoding a T6SS. To determine whether the T6SS is active *in vitro*, we assessed the presence of Hcp in culture supernatants using a polyclonal antiserum generated against a recombinant, purified and crystallized tag-free Hcp protein from *A*. *baumannii* AB0057.

**Fig 1:**
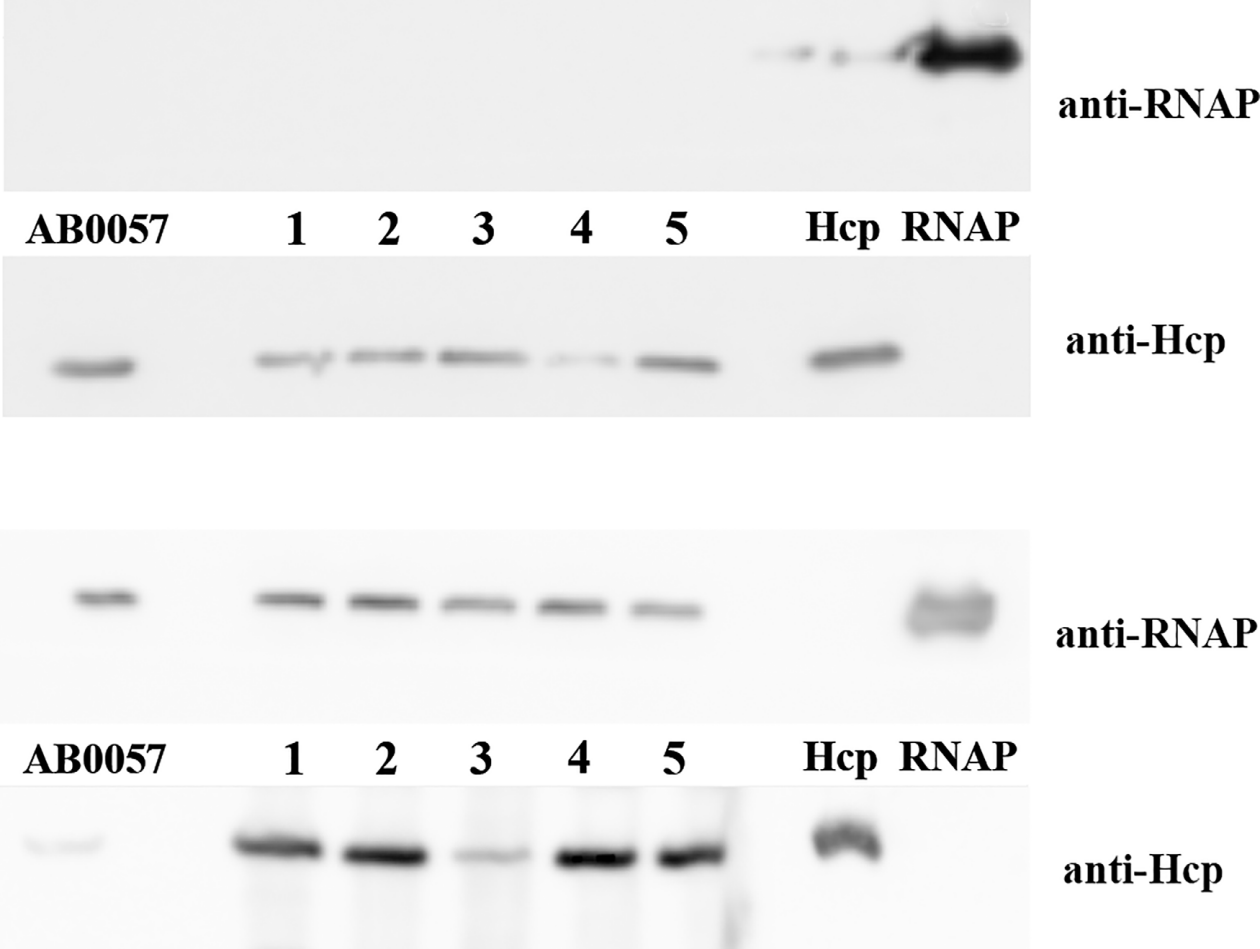
Inmunoblot analysis of Hcp secretion by nosocomial *A*. *baumannii* strains. Supernatant (top panel) and whole cell (bottom panel) samples prepared from *A*. *baumannii* AB0057 and other clinical isolates from diverse origins (1 from joint fluid, 2 from diabetic foot wound, 3 from skin lesion, 4 from urine and 5 from sputum) were probed with anti-Hcp and anti-RNAP polymerase as lysis control.

In order to test the functionality of this secretion system in other pathogenic *A*. *baumannii* strains we also studied other 5 clinical strains isolated at the Hospital Clínico San Carlos (Madrid, Spain). These clinical isolates were recovered from clinical specimens of patients being treated in different Hospital areas over an extended period of time (more than one year) to avoid duplication. The isolates, all of which had different antimicrobial susceptibility, were specifically selected from dissimilar biological sources: sputum, urine, skin lesion, joint fluid and diabetic foot wound. The strains were identified by matrix-assisted laser desorption ionization-time of flight (MALDI-TOF). Species identification was confirmed by amplification of *bla*_*OXA-51*_ gene and partial *rpo*B gene sequence. The Hcp sequences from the clinical isolates are identical to the one from the AB0057 strain. The Hcp protein was detected in all the supernatants studied, its secretion being less pronounced in the strain isolated from urine (Fig 1). Variations in the secretion of Hcp have been observed previously among other *A*. *baumannii* strains with some cultures showing strong secretion and others where the secretion is completely repressed in the tested conditions [23].

### Overall structure

Both constructs encoding the Hcp protein, with or without the His-tag, were crystallized in the same space group (P6). Despite the efforts to improve them, the crystals of tag-free Hcp were of poor quality and a complete X-ray diffraction data set could not be collected for this sample. Conversely, the C-terminal tagged Hcp crystals diffracted up to 1.55 Å, the highest resolution as yet reported for a molecule of this type. The unit cell dimensions were a = b = 87 Å and c = 128.8 Å.

**Fig 2:**
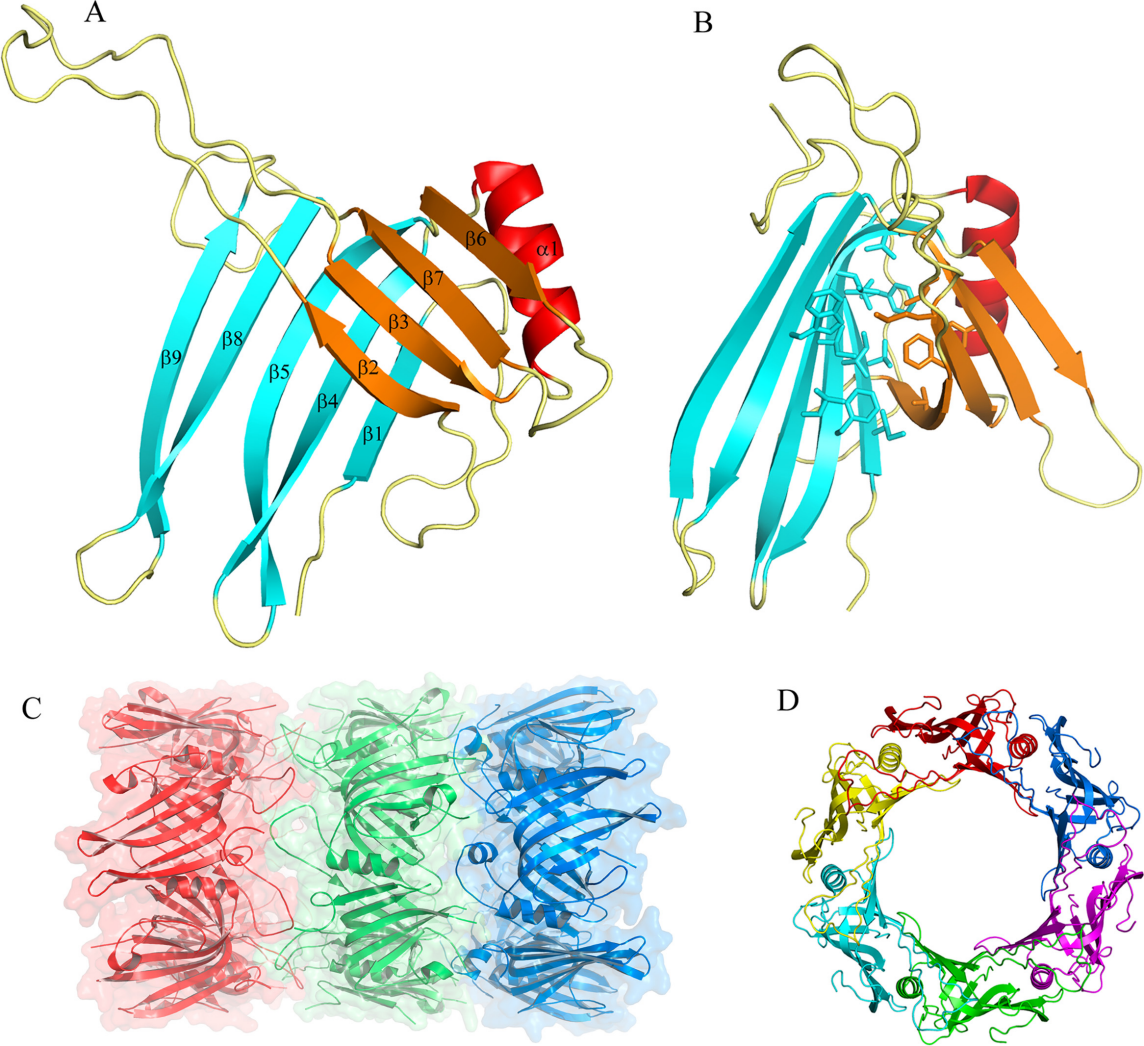
Overall structure of the Hcp from *A*. *baumannii*. (A) Cartoon representation of the Hcp structure whereby eight β-strands form a tight β-barrel, with one α-helix at one side, and an extended loop protruding more than 25 Å between strands 2 and 3. (B) Stick representation of the side-chain of hydrophobic residues forming the core of the β-barrel. (C) The Hcp hexameric ring. Cartoon representation of one hexamer generated by the 6-fold crystallographic symmetry. The α-helix of each monomer is placed almost parallel to the crystallographic axis. The diameter of the internal ring is 40 Å, while the external one is 80 Å. (D) Hexameric Hcp rings showing both, head-to-head (red-green) and head-to-tail (green-blue) packing.

The Hcp structure revealed a tight β-barrel domain (12 Å diameter) formed by two β-sheets that are comprised of 4 and 5 strands each (Fig 2A). The hydrophobic core of the barrel is formed by the residues: Val6, 29, 86 and 109; Phe8, 62, 90 and 128; Leu102, 106 and 130; Ile4, 88 and 104; Met60 and Trp32 (Fig 2B). The barrel is flanked by a 10-residue α-helix (from Ser70 to Gly80) and a loop, formed by the residues His34 to Asp59, that protrudes more than 25 Å from the core.

There are three molecules (0.1 Å RMSD between their Cα atoms) in the asymmetric unit that are packed in a down-up-up fashion, with a distance of 30 Å between chains A and B (head to head arrangement), 20 Å between chains B and C (head to tail) and less than 10 Å between chains C and a symmetry related chain A from the next unit cell (tail to tail). Chains A and B from the same asymmetric unit do not interact directly but rather, chain A interacts with a symmetry related chain B at the end of the extended loop, involving residues Thr43 to Val47. Accordingly, Thr43 is hydrogen bonded with Val47 and Ser45 forms two similar bonds with the same residue from the other chain. Thus, the area of the interface is only 578 Å^2^ and it is not significant for complex formation according to the PISA web server. Similarly, the residues from the N terminus of chain B interact with residues from the extended loop of a symmetry related chain C. The surface area of this contact is 358 Å^2^, which is not relevant for complex formation. Chain C interacts with chain A through their respective N terminal regions and furthermore, two hydrogen bonds are formed between residues Asp96 and Arg98 from chain C and Arg98 and Asp96 from chain A, respectively. Accordingly, the contact surface area is 400 Å^2^ and in agreement to PISA, it may be a crystal-packing artifact. In all the 3 chains the C-termini is exposed to the solvent, while the N terminal residues participate in inter-molecule interactions. The 5 extra residues (Gly-Pro-His-Met-Ala) that remain after His-tag cleavage might affect the crystal packing in the tag-free Hcp crystals and then the quality of their diffraction.

**Fig 3:**
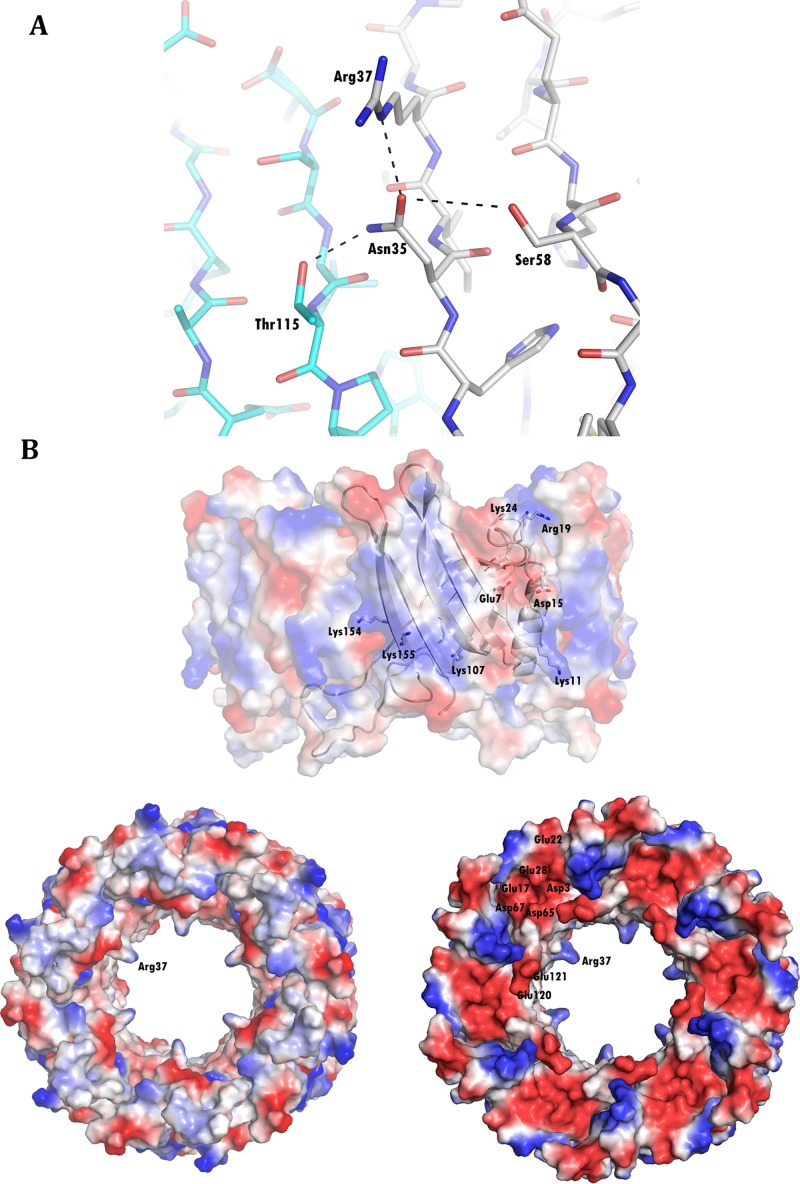
Key residues in the surface of the hexameric ring. (A) A hydrogen bond network stabilizes the position of Arg37, pointing to the central axis of the Hcp ring. Note the continuous β-sheet surface formed between neighboring chains (colored grey and cyan). (B) Electrostatic charge at the surface of the Hcp ring showing, in one of the edges, cavities with strong negative charge.

Using the 6-fold crystallographic symmetry, one hexameric ring can be generated from each Hcp chain (Fig 2C) interacting between them in both, head-to-head and head-to tail behavior (Fig 2D). The average contact area between intra-ring molecules is 1325 Å^2^, with 19 inter-chain hydrogen bonds. Among all the interfaces analyzed by the PISA web server in this structure, this is the only one that appears to play an essential role in complex formation. The inner diameter of this donut-shaped hexamer is 40 Å and the outer one is 80 Å. The 10-residue α-helix of each monomer is almost parallel to the crystallographic axis and it serves as a contact surface for the neighboring molecule. This helix interacts with residues Ser112 to Thr117 of β6 from the same chain, and with residues Val152 to Trp156 of a β-sheet (β8) from the symmetry related molecule. Pro71 faces Val152 and the aromatic ring of Tyr139, whereas Glu75 forms a hydrogen bond with the N atom of the Lys155 main chain. The side chain of Trp74 projects into the hydrophobic cavity formed by Leu66, Pro71 and Pro116 from the same chain, and by Trp32, Ile 36, Leu102 and Trp137 from the flanking chain. In addition, Trp74 interacts through a hydrogen bond with Glu126 from the same chain. The extended loop from the neighboring chain acts as a cap for the helix, interacting with their first residues. Moreover, hydrogen bonds are established between the O atom of the main chain of Gly80 and Cys77 with Lys40 and Gln38, respectively. Furthermore, Ser78 forms a hydrogen bond with the Nδ atom of His57and the main chain of Ser78 stacks against the side chain of Val55. The region from Ser112 to Glu120 (β6) interacts with residues Asn30 to Gln38 (β2) of the neighboring molecule. Accordingly, the six molecules form a continuous 24 β-strands surface and this β-barrel represents the inner surface of the ring. The Arg37 side chain points to the central axis of the hexagon, projecting from the barrel flat surface. A net of hydrogen bonds, involving Asn35, Ser58 and Thr115 from the next chain, maintains the particular position of Arg37 (Fig 3A).

The inner surface of the ring presents a slight negative charge, whereas on the external surface, regions with strong negative (around Glu7) or positive charges (around Lys154 and Lys24) can be detected. Both edges of the ring behave distinctly and while the side where the extended loop is exposed has repeated weak positive and negative charges, the opposite side exhibits cavities with strong negative charge. These latter regions are located around Glu17-Asp65-Asp67 at the N terminal region of the α-helix (Fig 3B).

**Fig 4:**
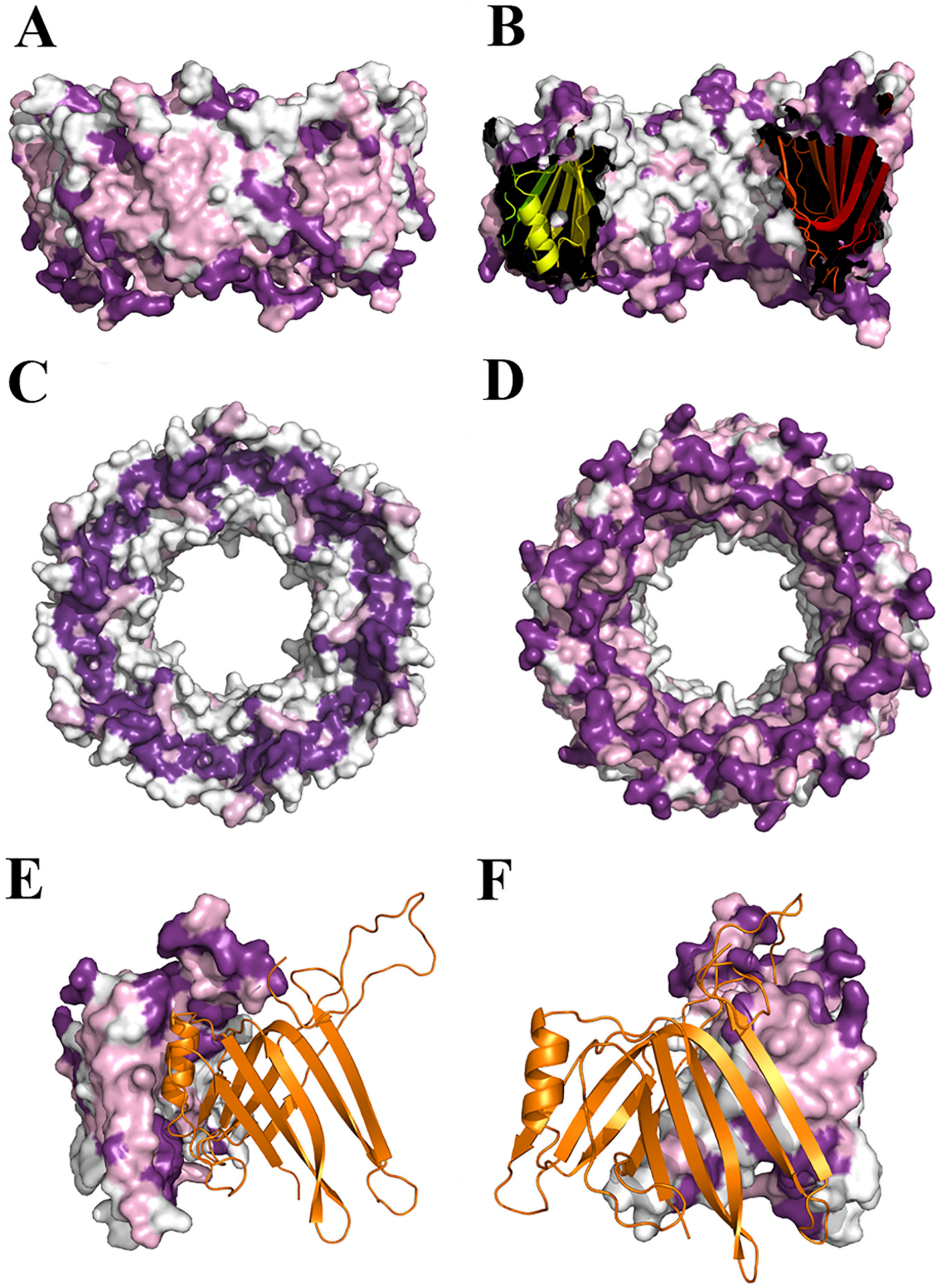
Conservation of residues in the Hcp surface. Degree of conservation of Hcp residues, the darkest colour representing the highest degree of conservation. Residues are moderately conserved in the external surface (A) and weakly in the internal (B), and they produce a circle of strong conservation at both edges of the ring (C and D). Strong conservation is also observed in the intra-ring interfaces (E and F).

In order to study the conservation of these residues, we aligned different Hcp sequences using BLAST and we plotted the degree of conservation in the Hcp ring structure using Consurf. The conservation among the surface residues is not uniformly distributed. The external side of the ring is characterized by a medium degree of conservation, while residues in the inner surface are poorly conserved. The highest degree of conservation was evident at the edge of the ring where the extended loop is exposed. However, a non-uniform pattern was observed on the other edge, with a thin circle of well-conserved residues surrounded by two regions of poorly conserved ones. The residues are also conserved in the intra-ring contact surface between monomers, showing higher conservation at the top and bottom of the α-helix, and at the residues that interact with the helix from the neighboring molecule (Fig 4). Interestingly, the residues interacting with Trp74, as well as this residue itself, are not conserved among the sequences analyzed, and none of the known structures have similar interactions in the middle of the helix.

### Oligomerization

**Fig 5:**
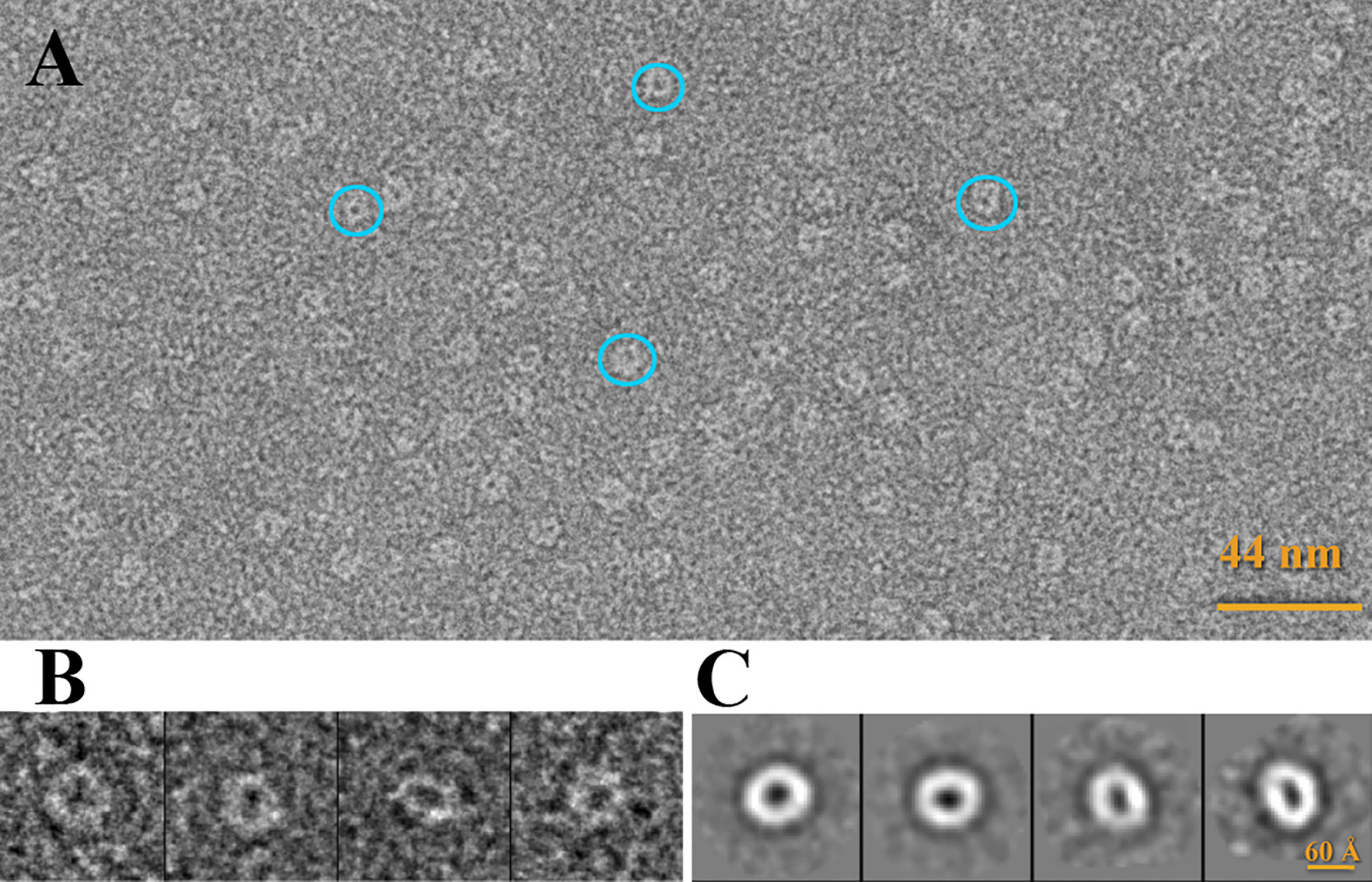
Electron microscopy of Hcp hexamers. (A) Electron micrograph of Hcp stained with 1% uranyl formate. (B) Single particles and (C) Reference-free 2D averages.

With the purpose of analyze the oligomerization state of Hcp in solution we performed sedimentation velocity experiments at different concentrations: 0.016, 0.2, 1, 2.5, 8 and 20 mg ml^-1^. The c(s) profile of the protein in these velocity analyses produced a single predominant peak at all the tested concentrations. The corresponding sedimentation coefficient for this peak was in the range of 5.0 to 5.6 ± 0.1 S. Moreover, sedimentation equilibrium experiments were carried out at two different concentrations and the experimental data fitted to a molecular mass of 114 ± 1 or 117.0 ± 0.5 KDa for Hcp concentrations of 1.0 or 2.5 mg ml^-1^, respectively, which correspond to the molecular weigth of six Hcp molecules (molecular weight of 18.8 KDa). At a lower concentration (0.02 mg ml^-1^) we analyzed the Hcp sample by negative-staining electron microscopy. The predominant geometry reflects a ring oligomer with dimensions (diameter: 60 Å) similar to those found in the crystallographic structure. Larger assemblies were not detected (Fig 5).

## Discussion

The Hcp proteins are considered to be molecular markers of a functional T6SS and they have been extensively used to evaluate the activity of this machinery in Gram-negative bacteria. We have demonstrated that Hcp is secreted to the culture medium by six different nosocomial strains of *A*. *baumannii*. Besides the diversity in their origin, the Hcp protein is identical in all these samples. The sequence is conserved among other *A*. *baumannii* strains, as confirmed with a BLAST search. Despite a few nucleotide substitutions there is no differences in the amino-acid sequences between the 25 different strains analyzed by this search. In particular, we have confirmed that the T6SS is functional in AB0057, a clinical multiresistant strain.

**Fig 6:**
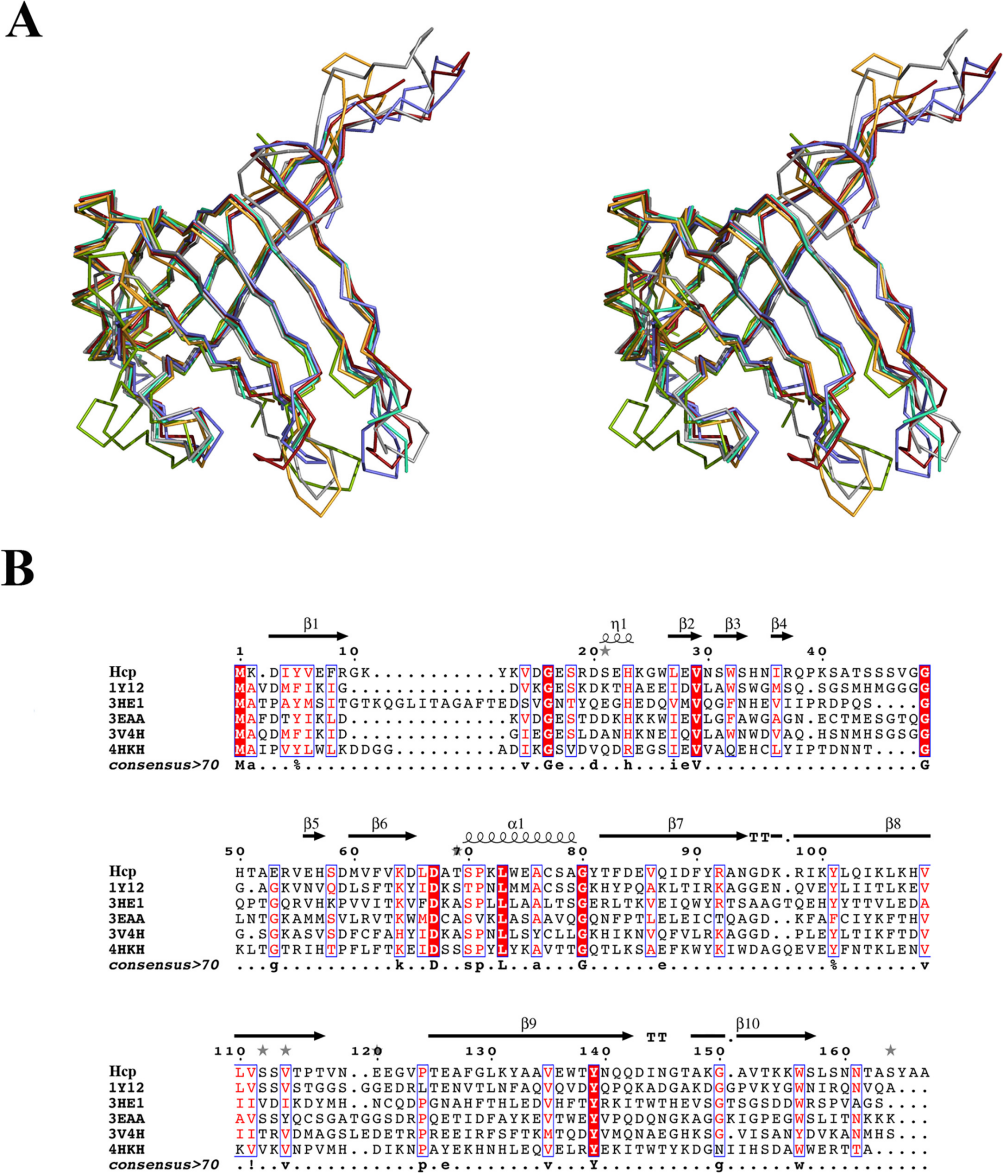
Structural superposition and sequence alignment of Hcp with other known Hcp structures. (A) Stereo view of Cα superposition of the structures: Hcp from *A*. *baumannii* (grey), Hcp1 (PDB entry 1Y12 in red), Hcp3 (PDB entry 3HE1 in green), EvpC (PDB entry 3EAA in blue), Hcp from *Yersinia pestis* (PDB entry 3V4H in cyan) and Hcp from EAEC (PDB entry 4HKH in orange). The tight β-barrel motif, the α-helix and the protruding loop are conserved in all six of these structures. (B) Sequence alignment showing the weak sequence identity among these proteins.

In order to gain a better understanding of the function and characteristics of this protein isolated from the *A*. *baumannii* AB0057 strain, we determined its X-ray structure. The overall structure of Hcp is formed by two β-sheets forming a tight β-barrel, flanked by a short α-helix and an extended loop, resembling other previously published structures of homologues proteins. Five Hcp structures have been determined by X-ray crystallography to date: Hcp1 (PDB code 1Y12)[4] and Hcp3 (PDB code 3HE1)[10] from *P*. *aeruginosa*; EvpC (PDB code 3EAA) [11] from *E*. *tarda*; the unpublished Hcp of *Y*. *pestis* (PDB code 3V4H); and more recently, Hcp from enteroaggregative *E*. *coli* (EAEC) (PDB code 4HKH)[12]. The sequence identity and similarity of Hcp from *A*. *baumannii* are 31 and 55% with Hcp1; 20 and 45% with Hcp3; 30 and 48% with EvpC; 26 and 56% with Hcp from *Y*. *pestis* and 20 and 47% with Hcp from EAEC. Despite the low sequence similarity among them, the structure of these proteins is almost identical (Fig 6): a Cα superposition with Hcp gives an RMSD value of 1.45 Å for Hcp1, 2.82 Å for Hcp3, 1.53 Å for EvpC, 1.22 Å for Hcp from *Y*. *pestis* and 2.16 Å for Hcp from EAEC. The Hcp-family fold is conserved. The main differences among these structures are seen in the length of loops connecting the strands, where Hcp shares more similarities with Hcp1, EvpC and the Hcp proteins from *Y*. *pestis* and EAEC than with Hcp3. This last protein has an insertion of 14 residues in the N terminus and it has been shown to belong to a different subgroup than Hcp1. This divergence was associated to certain specificity of Hcp proteins in *P*. *aeruginosa*, with different paralogues performing particular secretory functions. The stronger similarity in structure and sequence between Hcp and Hcp1 suggests that Hcp should acts in *A*. *baumanni* in a similar way to Hcp1 in *P*. *aeruginosa*.

The 6-fold symmetry established involves three Hcp molecules forming three hexameric rings. The inner diameter of this donut-shape hexamer is 40 Å and the outer one is 80 Å. These values were confirmed by electron microscopy, and they are similar to those described for Hcp1, Hcp3, EvpC and EAEC Hcp. The high degree of conservation among the residues involved in the hexamer formation emphasizes the importance of this ring in the protein´s biological activity. The conservation of residues at the outer face of the ring suggests that Hcp is likely to interact with other proteins during the formation or activity of T6SS, as proposed for Hcp1[4]. Interestingly the degree of conservation is lower at the inner face, on the basis of which we propose that the effectors transported through this ring, if any, may be specific to *A*. *baumannii*. In particular, the side chain of Arg37 may play a key role in this process. The guanidium group of this poorly conserved residue points to the center of the hole, while a network of other characteristic residues-none of them highly conserved- maintains this particular geometry. Interestingly, residue Ser31, a residue required for the secretion of the effector Tse2 [14], occupies a close position in Hcp1 from *P*. *aeruginosa*.

The most remarkable difference between the known Hcp structures is the crystal packing. Hcp1 forms a continuous nanotube with the rings interacting in a head-to-tail mode [4]. EvpC [11] and the Hcp from *Y*. *pestis* also form nanotubes, although their hexameric rings interact in a head-to-head mode. In the same manner, Hcp3 form dimers of hexamers but this latter structure does not adopt the form of a nanotube and dodecamers are isolated in the crystal [10]. Finally, two hexamers of Hcp from EAEC interact through each face in a head-to-head fashion but with a shift in frame of 0.5 hexamer. This uncommon packing might be induced by the two mutations incorporated in order to solve this structure [12]. Although the *A*. *baumannii* Hcp protein forms a nanotube, its packing is unique among these structures, as the 3 chains interact both head-to-tail and head-to-head within the same crystal.

The presence of only one peak in the size exclusion column (data not shown) and in sedimentation velocity experiments confirm that only one oligomeric state of Hcp exists in the conditions tested, and according to the sedimentation equilibrium the weight corresponds to that of a Hcp hexamer. Indeed, a hexameric ring is also the aggregation state observed in electron microscopy experiments. All these data suggest that the predominant oligomeric state of Hcp in solution is as a hexamer and hence the model of inter-ring interactions for this structure would be the result of the crystal packing in this particular condition. Similar to the observed by analytical ultracentrifugation and electron microscopy in other Hcp proteins there is no evidence of nanotube formation in Hcp from *A*. *baumannii*. Only Hcp1 associates in solution forming nanotubes; but this larger assembly appears exclusively after the mutation of residues, establishing disulfide bonds between adjacent rings [39]. However, the formation of Hcp nanotubes cannot be excluded in physiological conditions or in the presence of other T6SS proteins, as shown *in vivo* in EAEC where the proper assembly of the Hcp tube is controlled by other T6SS components. It was also observed in this organism that Hcp hexamers assemble in a head-to-tail behavior [40]. In A. baumanni a high degree of conservation is observed in both edges of the Hcp ring, and the surface charges complement each other between one edge and the opposite one, supporting the hypothesis of larger assemblies. More extensive functional studies are necessary to determine the importance of these conserved motifs and the oligomerization of Hcp from *A*. *baumannii in vivo*.
