# Correction: Crystal Structure of Hcp from *Acinetobacter baumannii*: A Component of the Type VI Secretion System

**DOI:** 10.1371/journal.pone.0136978

**Published:** 2015-08-26

**Authors:** Federico M. Ruiz, Elena Santillana, Mercedes Spínola-Amilibia, Eva Torreira, Esther Culebras, Antonio Romero

The following information is missing from the Funding section: This work was supported by FIS PI13/01471, BFU2011-24615 and CSD2009-00088 Spanish projects, and the Regional Government of Madrid (S2010/BMD-2353). F.M.R. was supported by an ESF/CSIC funded JAE-Doc Contract.
